# Stepped care and digital intervention service model design in the multidisciplinary sleep service

**DOI:** 10.1016/j.invent.2025.100830

**Published:** 2025-04-19

**Authors:** Sara Winter, Sara Crocker, Tricia Rolls, Deanne Curtin, Jessica Haratsis, Irene Szollosi

**Affiliations:** a Faculty of Medicine, University of Queensland, Herston, Brisbane, Queensland, Australia; b Psychology Department, The Prince Charles Hospital, Brisbane, Queensland, Australia; c Allied Health Research Collaborative, The Prince Charles Hospital, Brisbane, Queensland, Australia; d Sleep Disorders Centre, The Prince Charles Hospital, Brisbane, Queensland, Australia; e Metro South Addiction and Mental Health Services, Brisbane, Queensland, Australia; f School of Biomedical Sciences, University of Queensland, Herston, Brisbane, Queensland, Australia

**Keywords:** Humans, Insomnia, CBTi, Cognitive Behaviour Therapy for Insomnia, Digital transformation, Sleep initiation and maintenance disorders, Public health, Stakeholder participation, Health care costs, Primary health care, Patient care team

## Abstract

In order to ensure access to insomnia treatment in our public health environment of increasing patient acuity, increasing demand and health care costs, we need to innovate and implement systematised models of care to achieve better outcomes and efficiencies.

The design of a new Stepped Care treatment model in the multidisciplinary sleep disorders service with consumer and stakeholder engagement is described. Patients, their referrers and staff were surveyed to explore their views and preferences towards Stepped Care, including digital transformation. A consensus group workshop using the Nominal Group Technique was undertaken with the multidisciplinary team to develop the model of care.

The team endorsed a hierarchy of treatment steps beginning with digital intervention, group and trainee interventions as first line, escalating to more intensive 1:1 ‘upstream’ for higher acuity presentations. Referrer surveys highlighted the need for education in primary care settings about the availability of evidence-based internet treatment options. While few patients were aware of the availability of digital insomnia intervention, they were largely supportive of digital transformation. Barriers and risks to the Stepped Care approach were identified which informed the refinement of the treatment pathway.

Stepped Care treatment models offer adaptability and flexibility, allowing for adjustments in interventions based on patients' response to treatment, and preventing unnecessary escalation of care while reducing costs and improving efficiencies.

## Highlights

• Stepped Care models with internet intervention as first line are acceptable to stakeholders.
• Co-design methodology ensures high stakeholder satisfaction with service model redesign.
• Stepped Care models adapt to healthcare pressures for prevalent conditions like insomnia.
• Primary care education on access to evidence-based internet interventions is needed.

## 1 Introduction

Insomnia significantly impacts individual health and societal productivity, costing the Australian economy approximately $51 billion annually through direct and indirect costs (Deloitte Access Economics, 2021). Cognitive Behaviour Therapy for Insomnia (CBTi) is the gold standard treatment (Qaseem et al., 2016; Ree et al., 2017a) but access to qualified practitioners is limited (Meaklim et al., 2021; Ree et al., 2017b; Espie and Henry, 2023; Baglioni et al., 2023), with access to mental health support further constrained in rural areas (van Spijker et al., 2019; Sinclair et al., 2013). Despite its prevalence, most patients are prescribed pharmacological treatments, which have suboptimal long-term efficacy and potential for dependency (Miller et al., 2017). The Australasian Sleep Association (ASA) and the American Academy for Sleep Medicine (AACM) recognise that CBTi is a specialist intervention delivered by appropriately trained clinicians, who can also recognise and on-refer co-morbid mental and physical health conditions if indicated (Ree et al., 2017a; Edinger et al., 2021a). However in a local context, there are limited trained psychologists with sleep expertise (Meaklim et al., 2021). These pressures require implementation of systematised and innovative models of care, delegation to junior staff, and utilisation of time efficient (e.g., group delivery) and technological solutions designed to improve service efficiencies, effectiveness of care, and to maintain or improve patient reported experience and outcomes.

This study aims to develop a “Stepped Care” model within a public tertiary sleep service, and to understand stakeholder perceptions of insomnia treatments including digital options. Stepped Care involves a systematic approach to assessment, treatment, and monitoring, with interventions ranging from low-intensity, self-directed interventions to more intensive, specialist-led treatments. The key principle of Stepped Care is to provide the least intrusive and most effective intervention for each individual, with the option to step up to more intensive interventions if needed (Bower and Gilbody, 2005). This approach optimizes resource allocation, improves patient outcomes, and enhances healthcare delivery efficiency (Baglioni et al., 2023). This model often leverages digital, group, and trainee-led interventions as entry points, allowing for flexible, cost-effective care delivery that can be escalated if necessary (Espie, 2009; Darden et al., 2021; Espie et al., 2007). Digital CBTi interventions have shown effectiveness comparable to face-to-face treatments (Espie, 2009; Darden et al., 2021; Espie et al., 2012), making them a viable first-line option for patients with mild to moderate insomnia (Espie et al., 2012; Mahoney et al., 2022). Additionally, involving junior practitioners in delivering insomnia interventions not only meets increasing demand but also provides valuable training opportunities while maintaining high patient care standards (Mutsekwa et al., 2022).

This study engaged stakeholders and consumers to adapt the Stepped Care model to the service's specific context, with future work to focus on evaluating its implementation via the RE-AIM framework (Glasgow et al., 2019) with reference to patient clinical outcomes, patient/clinician service satisfaction, and service efficiencies.

## 2 Methods

This study employed a stakeholder-consumer-service co-design methodology using qualitative data collection methods to design the new model of care. This project has received ethics approval via the low or negligible risk (LNR) pathway with a waiver of informed consent from the Darling Downs Health Human Research Ethics Committee (LNR/2022/QTDD/88350). The protocol is listed with the Australian and New Zealand Clinical Trials Registry (ACTRN12622001086752).

### 2.1 Participants

Participants were multidisciplinary clinical team members of the Prince Charles Hospital Sleep Disorders Centre, consumers referred to the service between the dates of January 2021 and June 2023, and referrers (usually a General Practitioner) of the consumers referred during that time period. All multidisciplinary staff working within the service at the time of undertaking the consensus group workshop were invited to participate. Participant demographics for each phase of the project are presented in 3.1.1, 3.1.2.

### 2.2 Procedure

The project proceeded in three phases.

#### 2.2.1 Phase 1: consumer and stakeholder feedback

Consumer, referrer and staff attitudes about the proposed service model change was collected via online questionnaires. Consumers and their referrers were sourced from hospital administrative and clinical records of patients seen by the service between 1 January 2021 and 30 June 2023, resulting in 121 consumers and their 102^1^ referrers being mailed or emailed a link/QR code to the Consultation Hub questionnaire. Current clinical staff of the service (41 staff consisting of 12 medical doctors, 12 nurses, 13 scientists and 4 psychologists) were emailed a Microsoft Forms questionnaire to complete. Anonymous responses were collated by a researcher (SW) for analysis at the end of the consultation period.

#### 2.2.2 Phase 2: consensus group workshop

A modified consensus group workshop, using the Nominal Group Technique (NGT) (Delbecq et al., 1975) was undertaken in December 2022 with all clinical staff. Due to staffing, leave, and other clinical commitments, a total of 10 participants were in attendance. The full demographic breakdown of participants is presented in Table 2 in the Section 3.2.

The workshop was of 1 h duration, and was proactively facilitated by a single experienced clinician (SW) to ensure participants adhered to the NGT process. The purpose of the workshop was to elicit opinions on the proposed Stepped Care model including initial inclusion and exclusion criteria, and to gather expert advice on how this model should be modified for the local context including potential risks and benefits.

The workshops were completed as a hybrid meeting with participants present in person and online (Microsoft Teams). Workshop questions were emailed to participants in advance. Participants were advised that we were proposing a Stepped Care treatment approach for insomnia therapy, utilising a structure proposed by Espie (2009). Four proposed steps were identified as a potential ‘fit’ for our service context:

• Level 1: Digital insomnia therapy (“Managing Insomnia” by THIS WAY UP) (4 sessions) (Mahoney et al., 2022)
• Level 2: 1:1 manualised insomnia intervention (Sweetman et al., 2020) (adapted as necessary based on treatment formulation) delivered by a Psychology intern/trainee, supervised by the senior experienced Psychologist (4+ sessions as required)
• Level 3: Manualised group intervention (4 sessions) (Sweetman et al., 2020) co-facilitated by a Psychology Intern, supervised by the senior experienced Psychologist
• Level 4: 1:1 manualised intervention (adapted as necessary based on treatment formulation) with a senior experienced Psychologist with behavioural sleep medicine expertise (4+ sessions as required) (Sweetman et al., 2020)

The proposed Stepped Care structure posited that patients would be first triaged by the senior experienced Psychologist and assigned to the level most relevant to their needs using a collaborative decision-making model. It was expected that most patients would be assessed as suitable for entry to first-line manualised and online evidence-based digital treatment, with smaller numbers of patients assigned to the progressively more time, cost and expertise-intensive levels ‘upstream’.

Participants were asked the following 5 questions; “Do you have feedback on what the ‘Steps’ in the Model should be?”, “Do you have feedback on what the inclusion and exclusion criteria should be for each step?”, “What do you think is the right ‘flow’ through the Stepped Care Model (in-points and end-points)?”, “What benefits to you foresee from a change to a Stepped Care Model?”, “What risks to you foresee from a change to a Stepped Care Model and how might we mitigate these?”

These questions were answered in four stages, and preferred models were voted upon (McMillan et al., 2016; Mortensen and Holmes, 1983; Delbecq et al., 1986). See Supplementary Appendix A for more detail on the four stages of the workshop including the NGT process.

#### 2.2.3 Phase 3: presentation of final models

A collaborative in-service presentation was undertaken with the clinical team in February 2023 to present the themes that emerged from participant responses, as well as the results of the voting on the preferred service models. Final questions or clarifications were voiced, and planning for implementation was discussed.

### 2.3 Data analysis

Participant demographic details, as well as voting outcomes, are described using frequencies, percentages and range as applicable. Quantitative data collected via the Consensus group workshop were evaluated utilising inductive Thematic Analysis (Braun and Clarke, 2006; Xu and Zammit, 2020). Data was coded using iterative steps to ensure a robust approach, including 1) line-by-line verbatim transcription of the confidential responses from the Silent Generation phase across all 5 questions by SW; 2) responses were separated into succinct/separate ideas of a few words or a sentence depending on the nature of the response by SW. Where a response had several embedded ideas in the sentence, these ideas were separated for individual analysis; 3) independent data immersion by 2 independent coders (SC, TR) into data and line-by-line coding; 4) identification of preliminary themes and identification of supporting quotes by the independent coders; 5) review of generated themes and quotes by SW to refine and decide on final themes and quotes. Coding was performed manually. The decision was taken not to use software to analyse the data (such as NVivo) due to the relatively small data set.

Ideas generated and grouped through team agreement during the Round Robin and Clarification stages of the workshop in reference to questions 1 to 3 were transcribed during the workshop. These were not subjected to thematic analysis as these ideas were already synthesised by the group during the NGT process.

## 3 Results

### 3.1 Phase 1: stakeholder feedback

#### 3.1.1 Patients and referrers

Nineteen (15.7 %) patients and 6 (5.9 %) referrers completed the questionnaire. The mean age of patient respondents was 55.2 years (range 36–83) and the majority (79 %) were female. Referrers were predominantly female (83 %) had been practicing for an average 25.3 years (range 10–42) and estimated that approximately 25.3 % of their patients present with a sleep disorder (range 2–60 %). 100 % of consumers and referrers were living/working in a Major City based on Geographical Remoteness Area data^2^ (Australian Bureau of Statistics, 2021–2026) and 100 % of patients indicated they had access to the internet.

**Fig. 1:**
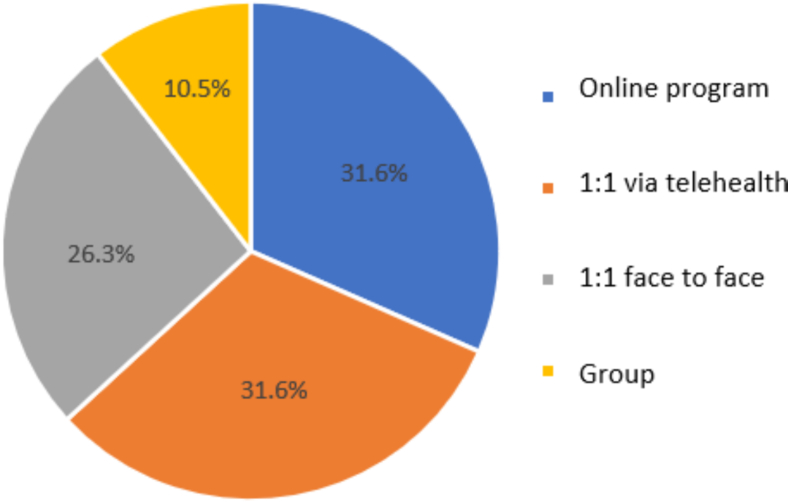
Patient preferred options for intervention mode of delivery.

Patients equally preferred receiving treatment through a self-directed intervention via an online program, and via one-on-one intervention with a therapist via telehealth (see Fig. 1). Approximately 15.8 % of respondents had used an online therapy for any problem in the past.

**Fig. 2:**
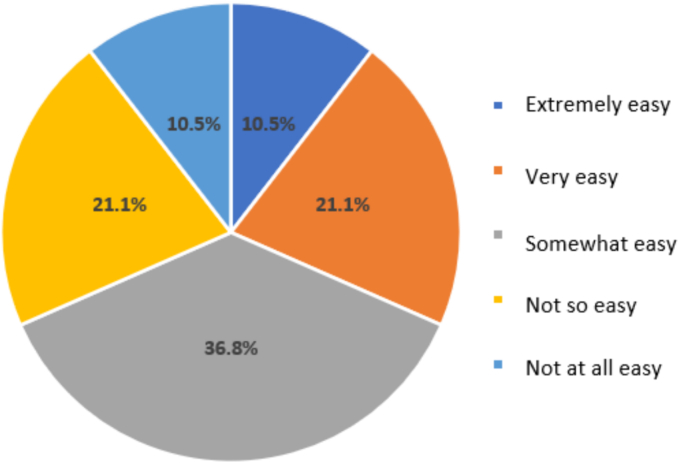
Patient perceived ease of accessing information and support via the internet.

Fig. 2 shows patients' perceived ease of accessing information and support via the internet with the majority indicating that this was ‘somewhat easy’.

When asked about their thoughts, comments or concerns if they were to be assigned to a digital intervention, 47.4 % of patient responses were coded as being ‘positively’ valanced (47.4 %) e.g. ‘*I would prefer online treatment as it is more convenient, given traffic congestion & work & family commitments*’; 26.3 % were ‘neutral’ e.g. ‘*no [concerns/comments]*’, and 26.3 % were ‘negatively’ valanced e.g. ‘*This would definitely NOT be my preferred way of treatment*’. Patient qualitative responses regarding group and digital treatments are presented in Supplementary Appendix B.

Approximately 50 % of referrers had prescribed some type of digital intervention, referencing mental health insomnia and pain management courses. Referrer qualitative responses are presented in Supplementary Appendix C.

#### 3.1.2 Staff

At the conclusion of the data collection phase, 16 (29 %) staff members had completed the questionnaire, with full demographic details presented in Table 1. The majority of staff participants were female (62.5 %), working full-time with the service (56.3 %), and 43.8 % were of the medical profession (31.3 % Sleep Physician, 12.5 % Advanced Trainee/Registrar).

**Table 1:** Demographic details of Staff respondents to baseline survey.

Demographics	Detail	N	Range
Profession	Sleep scientist	4	
	Sleep physician	5	
	Advanced trainee/registrar	2	
	Psychologist	2	
	Nurse	2	
	Other (health practitioner)	1	
Years practicing	Early career <1	1	<1–38
	1–10	7	
	11–20	3	
	20+	5	
Sleep Disorders Centre tenure (years)	<1	1	0–22
	1–5	7	
	6–10	2	
	11–20	5	
	20+	1	
Gender^a^	Male	6	
	Female	10	
Employment status^b^	Part-time	6	
	Full-time	9	
	Total participants	16	

a No participants nominated a non-binary or other gender option, or declined to disclose their gender.

b One participant did not respond to the employment status question.

A majority (81.3 %) of staff had heard of Managing Insomnia by This Way Up, although few (15.4 %) had previously prescribed the program to patients. 31.3 % of staff had prescribed some type of digital intervention in the past, referencing insomnia programs and mental health courses. A majority of staff members (93.8 %) indicated that they thought that digital CBTi was an appropriate and viable treatment option, albeit with some considerations in practice. Staff qualitative responses are presented in Supplementary Appendix D.

### 3.2 Phase 2: consensus group

A total of 10 staff members (24.4 %) attended the consensus group workshop. Full demographic details presented in Table 2. The majority of staff participants were female (60 %), had 1–5 years tenure with the sleep disorders service (60 %), and attendees varied across the medical, scientist, psychologist and nursing professions.

**Table 2:** Consensus group workshop participant demographics.

Demographics	Detail	N	Range
Profession	Sleep scientist	4	
	Sleep physician	3	
	Psychologist	2	
	Nurse	1	
Years practicing	Early career <1	0	1–35
	1–10	5	
	11–20	1	
	20+	4	
Sleep Disorders Centre tenure (years)	<1	1	<1–23
	1–5	6	
	6–10	0	
	11–20	2	
	20+	1	
Gender^a^	Male	4	
	Female	6	
Employment status	Part-time	5	
	Full-time	5	
	Total participants^b^	10	

a No participants nominated a non-binary, ‘other’ gender option, or declined to disclose their gender.

b Total number of participants who provided anonymous data/participated.

#### 3.2.1 Thematic analysis of silent generation responses

The key themes and sub-themes that emerged from the silent generation stage of the consensus group workshop are presented in Table 3. Representative de-identified participant responses are provided for each theme and subtheme for illustrative purposes in Supplementary Appendix E.

**Table 3:** Themes and sub-themes from Consensus Group Workshop.

Workshop grouping question	Themes and subthemes
Steps and Patient Flow	*As Proposed (digital → trainee → group → clin Psych)*
	*Reorder (digital → group → trainee → clin Psych)*
	Flexibility and patient centred
	Clinical Judgement/Assessment by Psychologist
Inclusion/Exclusion Criteria	*Flexibility and Patient Choice*
	Severity shouldn't preclude access to digital
	Guided by clinical interview
	*Criteria as outlined is Appropriate*
	Exclude significant co-morbidities to digital
	*More Consideration on Criteria is Needed*
Benefits	*Improved Access and Efficiency of Care*
	Improved access and equity
	Reduced wait times
	Increased throughput
	Reduced waste in resource/service provision
Risks	*Reduced Access/Efficiencies*
	Increase in Psychology demand
	Resource allocation inappropriate for clinical need
	*Gaps in Service Provision*
	*Unsuccessful Treatment/Sense of Failure*
	*Risk Management as Outlined is Thorough/No Changes*

Themes emerged from the 5 questions in relation to the proposed Stepped Care treatment approach, with responses around the Steps of the Model and Patient Flow questions clustering together. Therefore, themes and subthemes are presented in Table 3 relative to four grouping questions (Steps and Patient Flow; Inclusion/Exclusion Criteria; Benefits; Risks).

#### 3.2.2 Summary of Round Robin responses

The Round Robin stage generated a number of recommendations in response to each of the initial three questions asked of the clinical team. These responses are summarised below, and the raw responses as transcribed with the team during the workshop are presented in Supplementary Appendix F.

*Question 1: Steps in the Model*

The round robin responses were consistent with changing the order of the steps of the treatment approach to digital first, then group, then trainee and then senior psychologist. The team also identified additional roles for the psychology trainee, including the potential to lead the group intervention and to lead the check in for those assigned to the digital intervention.

*Question 2: Inclusion and Exclusion Criteria*

**Fig. 3:**
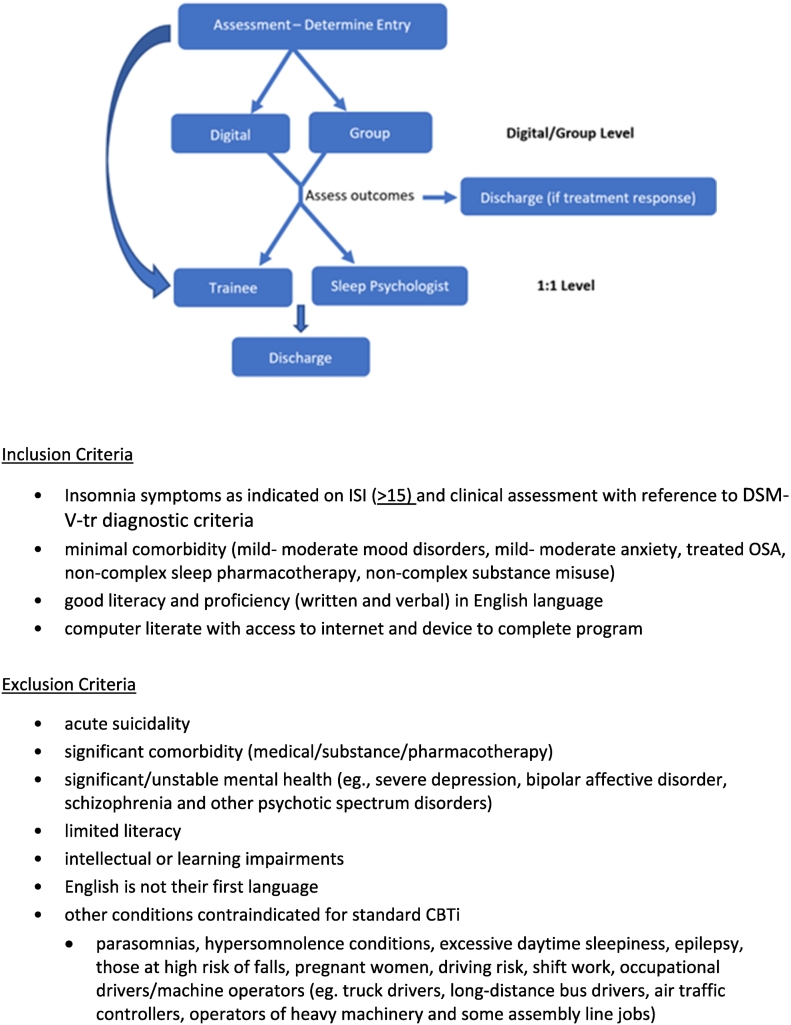
Stepped care model.

The team responses to questions of inclusion and exclusion criteria at each step of the treatment approach highlighted the need to maintain flexibility and decision should be patient centric. Inclusion criteria for entry to the four steps should be broad (for example, those with severe insomnia could still do the online program), however it would be more useful to focus on specific exclusion criteria (for example, mental health comorbidities) in determining assignment to steps. The team also suggested that differentiating who is suitable for trainee-led versus the clinical psychologist-led intervention would be an important triaging decision (see Fig. 3).

*Question 3: Patient Flow*

Participant responses around patient flow centred around initial in-points from Physician referral, recommending that Physicians could give information on the digital intervention to patients with the intention that they commence this option while they wait for a psychology appointment (documenting in the clinical record if they do so). Physicians could also provide sleep hygiene and basic sleep education advice as stepping stones to psychology referral. The team also suggested that those who don't respond to the digital intervention or the group should go to individual rather than through both before accessing individual treatment. The team also recommended that telehealth group options should be available to patients accessing the service.

#### 3.2.3 Summarising and voting

On summarising the outcomes from the round robin, the team identified three service model options for voting: ‘status quo’ referring to the current service delivery model (no change); ‘digital, then trainee, then group, then Senior Psychologist’; and ‘digital, then group, then trainee, then Senior Psychologist’. A total of 9 participants voted on their preferred model, perception of the ease of implementing each model, and perception of the ease of measuring each model option. Participants could provide the same score for preference/ease of implementation/ease of measurement for more than one model option. The results of voting are presented in Table 4.

**Table 4:** Consensus group voting.

Model	Preference (%)	Ease of implementation (%)	Ease of measurement (%)
Status quo	0	54 %	35 %
Digital, trainee, group, sleep psych	22 %	23 %	30 %
Digital, group, trainee, sleep psych	78 %	23 %	35 %
Number of staff casting votes	9	9	9

Participants indicated a clear preference for a stepped care treatment approach that commenced with a digital intervention as the first step, then group intervention, then trainee and finally individual therapy with a Senior Psychologist. No participants preferred to maintain the current service model (status quo). Participants indicated that maintaining the status quo would be easiest to implement (as no change would occur; 54 %), and that either status quo or the ‘digital then group’ model would be equally easy to measure (35 %, respectively).

### 3.3 Phase 3: presentation of final models

The final outcome of the consensus group was presented at a collaborate multidisciplinary team in-service, with the ‘digital, group, trainee and Senior Psychologist’ model identified as presenting with the best fit for the first round of implementation. The steps were refined to improve patient flow and reduce attrition for patients with incomplete treatment responses at the digital/group level, such that two ‘levels’ of intervention, with two ‘sublevels’ each articulated for implementation. Fig. 3 presents the stepped level approach agreed upon by the multidisciplinary team, along with the digital/group level inclusion and exclusion criteria.

Patients can be assigned to a digital or group level where they will complete either the digital intervention *or* group intervention before discharge (if treatment response is achieved) or escalation to a 1:1 level (trainee *or* Senior Psychologist) for those requiring further input. Escalation is based on clinician judgement of treatment response as well as unresolved insomnia symptoms based on the Insomnia Severity Index (ISI ≥15) (Bastien et al., 2001) and DSM-V-tr diagnostic criteria (American Psychiatric Association (APA), 2022). For those that escalate to a 1:1 level, content from the digital/group level is revised if necessary (but not repeated) and treatment is modified to target areas of concern based on the clinical formulation. For example, for patients presenting with nightmares an evidence-based treatment addition such as imagery rehearsal therapy may be incorporated (Aurora et al., 2010). Patients with comorbidities for which sleep restriction is contraindicated (e.g. bipolar disorder) would have this component of the treatment adapted in the 1:1 treatment (Edinger et al., 2021b). Note that patients who are identified at assessment as not suitable for the digital or group level (see inclusion and exclusion criteria), or who decline this level of treatment despite clinical suitability, can be escalated to 1:1 intervention, bypassing the digital/group level. The team identified that the stepped care treatment approach may be further refined by an iterative process during implementation. At the conclusion of the in-service, the team was satisfied with the model and reached agreement to commence implementation of the service model change from 1 July 2023.

## 4 Discussion

A heath care system under pressure requires services to re-imagine, transform and optimise their models of care to help more patients overcome highly prevalent conditions within existing resources. Sleep disorders are common and costly, and psychological intervention within a multidisciplinary sleep service contributes to service efficiencies and effectiveness, particularly for insomnia which is the most prevalent of the sleep disorders. This service model re-design to develop a Stepped Care model incorporating digital, group and trainee level treatments as first line, utilised consumer and stakeholder engagement throughout to ensure that the new service model was robust, safe and acceptable to all stakeholders. The Stepped Care model was developed and implemented in the sleep service alongside a “Direct to Psychology” referral pathway innovation described elsewhere (Winter et al., 2024).

Stepped care treatment approaches offer inherent adaptability and flexibility, allowing for adjustments in interventions based on patients' responses to treatment, changes in their circumstances, and emerging evidence. This flexibility enables clinicians to tailor interventions in real-time, optimise treatment trajectories, and prevent unnecessary escalation of care. Studies have demonstrated the efficacy of stepped care models, particularly in mental health settings, showing comparable outcomes to traditional approaches while often reducing costs and improving service efficiency (Mongelli et al., 2021; Cross and Hickie, 2017). This is especially relevant in a post COVID-19 health setting, where patients are more acclimatised to accepting telehealth and online digital treatments from the relative safety of home, versus attending hospital systems face to face (Blignault et al., 2022).

Model of care innovations that feature stepped-care principles have been found to be positive determinants of patient satisfaction and service efficiency (Espie et al., 2012) highlighting the benefits of digital CBTi modalities as a treatment option within sleep psychology services. Precedence exists in other areas of tertiary healthcare that have embraced digital and adjunct interventions, particularly in services relating to the aged and palliative or rehabilitation care, and chronic disease management (Kaambwa et al., 2017; Rushton et al., 2021; Walklin et al., 2023). We anticipate similar patient care benefits including ease of access, treatment adherence, and optimised patient safety (Dahlberg et al., 2020; Ngwatu et al., 2018; Osterloh et al., 2023). A majority of patients, referrers and staff indicated a willingness to accept digital interventions as part of the treatment model, although a lack of knowledge about the availability of these programs, challenges with digital literacy, a perception of reduced therapeutic benefit from digital treatment, and expectations about suboptimal treatment adherence were identified by stakeholders as key potential limitations which could affect uptake of this treatment modality. This suggests that patient, referrer and staff education about anticipated benefits and potential limitations (and strategies to ameliorate these) is critical to undertake alongside any service model re-design.

The resulting stepped care model presented here is a variation of the traditional version described by Bower and colleagues (Bower and Gilbody, 2005), where typically all patients start with the lowest intensity treatment level. However, Bower and colleagues note that in “*stepped care more intensive treatments are reserved for people who do not benefit from simpler first-line treatments, or for those who can be accurately predicted not to benefit from such treatments*” (p11). In the case of CBTi, there are known contraindications for some aspects of the treatment for individuals with known comorbidities (e.g. sleep restriction is contraindicated for those with bipolar disorder or seizure disorders) (Edinger et al., 2021b). Therefore, we can reasonably predict that patients with these comorbidities should be excluded from the group/digital level as they will require closer monitoring and adaptation to the sleep restriction component to the treatment. Furthermore, Bower et al., acknowledge the opportunity for patients to escalate to individual therapy should they decline lower intensity treatment. While balancing the efficiency assumption (as outlined by Bower et al) against treatment efficacy and the acceptability assumption, we believe we have developed a model, through stakeholder engagement, that provides the best opportunity for efficient, effective and acceptable care.

Baglioni et al. (2023) provide a useful guide for implementing a stepped care model for management of insomnia to prevent the risk of suboptimal care being offered, and to maintain high clinical standards when non-clinician led interventions are delivered. Adhering to the following principles has ensured the resulting model described here will be appropriately implemented; (a) only evidence-based CBTi is offered within the model; (b) clinicians involved in the model are suitably qualified to offer CBT, and have (or are receiving) further training in CBTi; and (c) the professionals involved in care provision but not included in the model (such as multidisciplinary staff members and referring practitioners) have good knowledge of the model in order to support allocation to the appropriate intervention step. While the Stepped Care treatment approach offers a flexible pathway to delivering interventions for sleep disorders, it also presents with limitations. Implementing the Stepped Care intervention requires significant resources in terms of staffing, training, and infrastructure within the hospital, including access to treatment rooms and resources by multiple clinicians often simultaneously. Addressing concerns about the increase in demand for psychology was paramount to the acceptability of this proposed model. We believe this is addressed within the inherent framework of the stepped-care hierarchy, which includes accessing an adjunct workforce of trained and supervised intern psychology clinicians to assist the broader sleep psychology team in managing patient needs.

Disparities in access to healthcare services may hinder individuals from progressing through the steps, especially in regions with limited access to internet services or for individuals that have low digital or health literacy. This may result in patients needing to access 1:1 treatments in person, at increased financial and logistical costs, who would otherwise have been suitable for a self-guided online intervention.

While the sample size for the consensus group was in line with empirical data on the sample size required to reach saturation for thematic analysis (Hennink and Kaiser, 2022), the response rates to our engagement surveys were low (6 % for referrers, 16 % for patients and 29 % for staff). This raises the possibly of response bias such that non-responders were not in favour of changing the model of care. Low response rates for Primary Care/General Practitioners to surveys is a known challenge (Templeton et al., 1997). However, there is emerging evidence in the literature regarding response rate and survey generalisability suggesting no substantive relationship between non-response bias and response rate (Hendra and Hill, 2019). All responders were located in a metropolitan area, and therefore the conclusions also need to be drawn within this context. While acknowledging the potential for bias within the responding, the current study also attempts to gather information across sources to triangulate preferences for model of care change and notes general consistencies including an openness for digital therapy and alternative service delivery modalities. The acceptability of the model for those participating in the new model of care will be tested in a planned post-implementation evaluation.

Further challenges exist in successfully engaging and retaining patients throughout the self-guided, digital treatment level. Factors such as motivation, stigma, and logistical barriers may impede individuals from actively participating in treatment, leading to suboptimal outcomes. We plan to address these potential challenges by securing patient participation with bi-monthly clinician ‘check-in calls’ to address barriers to engagement, and make administrative changes to triage and intake processes to flow more participants to the digital arm if indicated.

## 5 Conclusion

Streamlining Sleep Psychology interventions into a Stepped Care treatment approach including digital interventions as first line will allow patients to access the appropriate level of care based on their level of acuity and clinical presentation, with the model being flexible enough for patients to be expedited to higher levels of care based on treatment response. We posit that this will increase acceptability and access to sleep psychology treatment, thereby improving patient outcomes. Post-implementation evaluation is planned for this service model re-design with reference to patient clinical outcomes, patient/clinician service satisfaction, and service efficiencies pre- and post-service change.

## Funding

This project is supported by funding from a Metro North Allied Health AH-TRIP (Allied Health Translating Research Into Practice) start-up grant and the Chief Allied Health Officer (OCAHO) Health Practitioner Research Scheme; 10.13039/100010230Queensland Health.

## Declaration of competing interest

The authors have no declarations of interest to declare.

## Glossary

CBTi: Cognitive Behaviour Therapy for Insomnia
NGT: Nominal Group Techique

## Appendix A Supplementary data

Supplementary material Image 1

## Footnotes

1 102 as some referrers had referred multiple patients.

2 Australian Statistical Geography Standard (ASGS) Edition 3 (2021) defines remoteness areas within Australia based on geographical access to services. It divides Australia into five classes of remoteness, representing Very Remote Australia, Remote Australia, Outer Regional Australia, Inner Regional Australia and Major Cities of Australia. Access to services is indexed by the Accessibility/Remoteness Index of Australia Plus (ARIA+) which is the official classification of remoteness used by the Australian Bureau of Statistics (ABS).
