# Supplementary material for: Stepped care and digital intervention service model design in the multidisciplinary sleep service
Source: Internet Interv. 2025 Apr 19;40:100830. doi: 10.1016/j.invent.2025.100830 (PMC12083906; doi:10.1016/j.invent.2025.100830)
Supplement: Supplementary file 1 — Supplementary material [file mmc1.docx]

**SUPPLEMENTARY MATERIALS**

**Supplementary appendix A**

*Stage 1: Silent Generation*. Participants had 20 minutes to individually write down their responses to the five questions in phases or statements.

*Stage 2: Round Robin.* The facilitator (SW) conducted a round-robin feedback where all participants read out their responses, until no new ideas were forthcoming. All ideas are written on a virtual whiteboard for review. The first three questions in each workshop were prioritised at this step, with the two final questions on benefits and risks summarised and evaluated from the written responses (silent generation) post-workshop.

*Stage 3: Clarification.* The facilitator encouraged group interaction including questions and comments on the listed ideas. Similar ideas, on agreement from participants, were grouped together. Example service models based on the generated ideas were developed collaboratively with the group. The facilitator hosted a discussion around the generated ideas and example service models to ensure clarity and to enable group members to make an informed decision when it came to voting at Stage 4.

*Stage 4: Summarising and Voting*. Based on results of stages 1-3, the results were summarised and service model options were decided upon. Participants are asked to individually vote on their preferred models on a scale from 1 to 5 (1= least preferred model/5=preferred model). It was possible for participants to prefer more than one model equally. The preferred model/s (or the option with the highest score from 1-5) for each participant was assigned a value of 1 and these votes were tallied for a total score indicating overall votes for the team’s preferred model. Where participants indicated that they had an equal preference for more than one model option, each option was assigned a value of 1. Participants were also asked to individually rank how achievable each example service model would be to *implement;* and also to *measure* the outcomes of each service model; from 1 to 5 (1=extremely difficult implement to 5=easy to implement/ 1=extremely difficult to measure to 5=easy to measure). Again, the model assigned the highest value from 1-5 for each participant for each of these criteria were assigned a value of 1 each for to be tallied for the overall ‘votes’ for each model. This stage of the process was confidential.

**Supplementary appendix B**

The vast majority of patient responses were favourable to group therapies and digital CBTi treatment modalities, having either tried it previously with success or were open to allocation to this treatment option in future;

*Having gone through the group program in person at [hospital] with psychologist I am MOST enthusiastic about the sleep program offered in any way it is delivered and recommend it for people with sleep issues.(2)*

*I'm a one on one therapy fan but, as I've never done online therapy before, I wouldn't knock it until I'd tried it so, yes I'd give it a try.(6)*

*I’m happy to try it.(13)*

*I have no issues regarding online programs. Apart from the real human interaction, they are basically identical but way more practical and efficient than in-person.(18)*

Of the responses indicating a less favourable impression of digital interventions, the majority indicated low confidence in managing the practical aspects of the technology, followed by a perception of reduced anticipated therapeutic benefit from digital intervention, and concerns about online cyber security.

*Really prefer one on one in person as I feel more at ease talking in person....just feel more comfortable as I am not competent with using the internet.(7)*

*Not sure I could manage it (11).*

*I struggle with my capacity to learn when it comes to online platforms, it just doesn’t seem to work for me. I seem to get better results from in person clinic platforms, either one on one or in a group. (14)*

*It would have to be a passive learning exercise for me.(9)*

*I am extremely reluctant to sign up for any form of social media involving cameras, personal revelations or any other details I am not prepared to share with the world at large. I am not on Facebook and have no intention of joining.
Privacy is very important to me to the extent that I have even resisted joining Zoom. Call me paranoid if you wish but I have read too many horror stories of people having their identities stolen or being targeted by hackers.(9)*

One response indicated previous challenges with a CBTi program delivered face to face due to significant medical co-morbidities, and therefore did not expect a different outcome from a digital treatment;

*Because of my specific medical issue i couldn't relate to it any more than when i was being seen by a sleep psych in person and using the same methods over and over trying to resolve the situation.(20)*

**Supplementary appendix C**

While referrer exposure and understanding of digital CBT therapies was limited, those who were familiar with this treatment modality indicated that they had prescribed it to their patients and found it acceptable and helpful, although suitability for this treatment modality was limited to patients who were computer literate and had access to digital resources.

*[digital therapies are] Helpful in a select group - computer literate and motivated (1)*

Other feedback from referrers varied in terms of their expectations and experience with online therapies, from an impression that face-to-face is superior for psychoeducation delivery;

*The quality of the psychoeducation is not as good as face - to face (5)*

to a positive impression particularly as a treatment option that allows timely access;

*Patient feedback was promising. She was happy to be offered that option and I was glad that psychotherapy for sleeping was addressed at a time where the patient needed it most (6)*

and an interest in learning more;

*I am unsure about the details of programs. I would like to have some information (2)*

**Supplementary appendix D**

Staff were supportive of digital insomnia intervention albeit with the requirement of clinical oversight of patient’s assessment and progress, and highlighted the importance of patient choice in the process;

*[I’m]…just wanting a trained person to review and intervene when required.(15)*

*…would be happy to supervise them though the online program, use as an adjunct/between sessions.(11)*

*I would prefer initial f2f evaluation and management and selective/suitable patient can then go onto online CBTi.(6)*

*Supportive for patients who understand technology, they need to have an option to then have face to face sessions if online is not working for them.(9)*

Staff did express some concern about the likelihood of patient’s adhering to the online program and the importance of digital literacy.

*I don't feel like the patient would follow the online program.(3)*

*I think these programs have a place in clinical practice…The main issue is working out patient selection as some patients may be less IT literate, more comfortable with face-to-face etc. The other issue is ensuring that patients enrolled in an online program are progressing and completing the CBTi program. (4*)

**Supplementary appendix E**

De-identified participant responses to the consensus group questions are provided for each theme and subtheme for illustrative purposes*.* Each response is identified by a response number presented in the order that the response was transcribed into the larger dataset from the Workshop. Each theme and subtheme was presented in order of preponderance of responses to provide an indication of the strength of each theme. However, it should be noted that the importance placed on each theme was also considered in light of the questionnaire (Phase 1) and consensus group voting.

When considering appropriate “Steps” in the Stepped Care Model, participants did not identify any additional or alternative Steps required. That is, two levels of 1:1 intervention – with a trainee or the Psychologist with behavioural sleep medicine experience; one group intervention level; and one digital intervention level were sufficient. Responding to the question of the appropriate Patient Flow for patients through the Psychology service via these steps revealed two predominant themes: As Proposed (digital, then trainee intervention, then group intervention, then experienced Psychologist intervention), and Reorder (digital, then group, then trainee, then experienced Psychologist).

***As Proposed (digital 🡪 trainee 🡪 group 🡪 sleep Psych)***

A key theme around the flow through the Stepped Care Model was endorsement of the order of flow proposed in the Workshop. That is, that digital intervention should be the entry level step to the model, then 1:1 intervention with a trainee, then group intervention, then 1:1 with the Senior Psychologist.

*Currently proposed 4 step model appears to be a sound starting point (16)*

*I think as per the presentation, the steps proposed would work best (43)*

*Digital then trainee may result in better results faster than digital to group (44)*

***Reorder (digital 🡪 group🡪 trainee 🡪 Clin Psych)***

The second key theme to emerge was to reorder the flow, with group intervention to be second step after digital intervention, rather than 1:1 with the trainee Psychologist. This theme had two associated subthemes; Flexibility and patient-centred, and clinical judgement/assessment by experienced Clinical Psychologist.

*Prefer digital 🡪 group 🡪 psychology intern 1:1 🡪 Psychologist 1:1 (19)*

*Flexibility and Patient Centred*

A clear subtheme was a preference for a flexible and patient-centred approach to decision making about where patients are assigned within the steps of the model. Responses highlighted the importance of patient agency in consenting to and requesting change to the different levels/steps. There were suggestions for additional baseline information that all patients could receive regardless of entry step, such as baseline sleep hygiene information or information on the digital program for self-referral if they wish.

*Include that (sic) levels are suggested, however, pts[sic; patients] are free to consent/request change to any level(9)*

*In at any point, however if waiting to have an appt with intern, group or snr Psychologist then commence online in background? (40)*

*Clinical Judgement/Assessment by Psychologist*

Participants identified that decisions regarding patient assignment to the different steps in the model should be grounded in clinical judgement based on a thorough assessment by the experienced Senior Psychologist.

*Review by advanced clin psych. to assess appropriate level (3)*

*I think the order is probably best decided from clinical outcomes and ability to deal with complex issues (17)*

When considering appropriate Inclusion and Exclusion Criteria for the Steps within the model, three key themes emerged; Flexibility and Patient Choice, Criteria as Outlined is Appropriate, and More Consideration on Criteria is Needed.

***Flexibility and Patient Choice***

Participants highlighted the importance of flexibility with inclusion and exclusion criteria, with a focus on patient agency and choice in the step/s to which they are assigned in the model. Within this theme, two subthemes were evident; severity shouldn’t preclude access to the digital option, and all decisions are guided by the clinical interview.

*Criteria should be flexible for reasons such as distance/preference etc. (24)*

*Patient centric (29)*

*Severity shouldn’t preclude access to the digital option*

Participants suggested that patients should be able to access the digital intervention, even with increased clinical complexity/severity, but that closer clinician follow-up may be required for this cohort of patients.

*If pt who is severe wants to access online, should be available (sic) with closer f/up (22)*

*Guided by the clinical interview*

Participants also recommended that decisions regarding inclusion or exclusion to the different steps within the treatment approach should be guided by the formal clinical interview and clinician judgement, as should screening for higher risk presentations and circumstances (eg. driving vigilance risk).

*As per initial consult as determined in interview (28)*

*Screen for red flag symptoms eg. driving (69)*

***Criteria as Outlined is Appropriate***

A theme relating to participants being satisfied with the inclusion and exclusion criteria outlined in the workshop became evident, with one associated subtheme; exclude significant co-morbidities to the digital option.

*Appropriate (as outlined) (32)*

*Exclude significant co-morbidities to the digital option*

A subtheme within this theme related to a recommendation that significant comorbidities (such as significant mental health presentations) should be considered exclusion criteria for the digital intervention.

*Exclude significant co-morbidities ie. mental health (27)*

***More Consideration on Criteria is Needed***

Finally, when considering inclusion and exclusion criteria, a theme emerged suggesting that the criteria for inclusion and exclusion may need further consideration, with participants questioning how differentiation would be made between those suitable for individual vs group vs digital intervention.

*Inclusion criteria for individual therapy and intern vs group may require more consideration – ie/ should pt be directed to group in first instance? (25)*

When considering the Benefits anticipated if the Stepped Care treatment approach was implemented in the Psychology service, a key theme of Improved Access and Efficiency of Care emerged.

***Improved Access and Efficiency of Care***

Participants anticipated that implementation of the treatment approach would improve equity and access to evidence-based care for patients, and would improve service level metrics such as reduced wait times. Four subthemes emerged; Improved access and equity, reduced wait times, increased throughput, and reduced waste in resource/service provision.

*Faster access to sleep psychology input for all (54)*

*Improved access and equity*

Participants expressed a clear expectation that patients would experience improvements in equitable access to evidence-based care.

*Better equity to access to treatment (ie. rural/remote) (48)*

*Quicker access to help as well as different modes of help which accommodates for all types of people who respond better to different types of therapy (60)*

*Reduced wait times*

Furthermore, participants anticipated that service level outcomes such as wait times would improve with the model of care change, particularly for those who require more urgent treatment.

*This will hopefully improve patient flow through the system and reduce wait times especially for patients with severe symptoms (56)*

*Increased Throughput*

Increased and more efficient throughput from intake to the sleep Psychology service to discharge was also an anticipated benefit of the Stepped Care Model.

*More throughput of patients (57)*

*Reduced waste in resource/service provision*

Finally, participants anticipated that there would be reduced ‘waste’ in resource allocation within the system within a Stepped Care approach, which would benefit those patients requiring more intensive intervention, and allow them to access appropriate care.

*Reduced one on one time with senior Psychologist for those who don’t necessarily require it = increased efficiency, speed of access to care (50)*

*Less wasted resources/appt that are not needed (58)*

When considering potential Risks for consideration and management if the Stepped Care treatment approach was implemented, participant responses clustered around four key themes; Reduced Access/Efficiencies, Gaps in Service Provision, Unsuccessful Treatment/Sense of Failure, and Risk Management as Outlined is Thorough/No Changes.

***Reduced Access/Efficiencies***

Whilst participants noted several potential benefits to implementation of the Stepped Care treatment approach, including themes of access and service efficiency, participants also noted that there was a risk of reduced access and efficiencies if certain considerations were not taken into account and managed within the model. Two subthemes emerged on these considerations; increase in Psychology demand, and resource allocation inappropriate for clinical need.

*Possible delay in some difficult to manage cases to seeing level 4 Psychologist (66)*

*Increase in Psychology demand*

Participants questioned whether the service model change may inadvertently result in an increase in referrals/demand (particularly as the service model became known to external referrers), and that the reliance on the trainee workforce in terms of continuity of patient care needs to be considered as the duration of each trainee placement is time limited (6 months duration).

*Overwhelmed with patients and follow up (70)*

*How long does 1:1 take as in 5 sessions, 3 months? Will this implicate change over of interns or spill into snr[sic. senior] if no intern to take over? (67)*

*Resource allocation inappropriate for clinical need*

Participants also highlighted a risk of patients being filtered into levels that do not match their clinical need given that patient choice will be included in the allocation decisions. Participant responses were weighted towards a risk of patients with less complex presentations requesting 1:1 intervention when they would be likely to respond well to group or digital intervention, which may impact on access to care for patients requiring the 1:1 treatment modality.

*Pts requesting level 4 despite not necessarily requiring it (when giving pts preference/choice of level) (63)*

***Gaps in Service Provision***

Another theme emerging from the question of potential risks was that there may be gaps in service provision which may result from the way the model is structured and implemented. Identified potential gaps were that patients may require additional support or information on how to access to the digital intervention website, resulting in an imbalance of appointment types with patients being funnelled into one intervention type (step) over others. Lastly, that group therapy may be a barrier to treatment effectiveness, as group attendance rates can be lower than 1:1 intervention types (20, 21).

*Balance of appts eg. is everyone getting funnelled into digital and no appts booked for psych or vice versa (62)*

***Unsuccessful Treatment/Sense of Failure***

Participants identified that a risk of the treatment approach was that patients who did not respond to a less intensive level of intervention (eg. group or digital) may develop a sense of failure or reduced confidence in the treatment efficacy. Participant themes suggested this could result in drop out if they were otherwise suitable for escalation to another step in the model.

*Patients may drop out after digital if not successful and not wish to continue (71)*

***Risk Management as Outlined is Thorough/No Changes***

Finally, a theme emerged around participants satisfaction that the treatment approach accounted for potential risk, and had adequate provisions built into the treatment framework to manage these.

*I think your risk assessment as outlined is thorough – no further to add (61)*

**Supplementary appendix F**

**Question 1**: Do you have feedback on what the “Steps” in the Model should be?

Digital, group, intern, and/or clin psych

Intern led group program

Intern led check in with digital therapy

**Question 2**: Do you have feedback on what the inclusion and exclusion criteria should be for each step?

Severe on ISI could do online program

Maintain flexibility

Keep inclusion criteria broad, then some specific exclusion criteria (esp regarding MH comorbidities)

Differentiating who goes to intern vs clinician group

Patient centric

**Question 3**: What do you think is the right ‘flow’ through the “Stepped Care Model” (in-points and end-points)?

Physician gives information on this way up for pt to self-initiate while waiting for psychologist

Those who don’t respond to online/group to individual?

Physician documents if they have been suggested this way up in referral to psychology

Physician recommends sleep hygiene and basic sleep psyched.

Telehealth group options (eg. once or twice a year a telehealth group)
